# Association of thyroid autoimmunity with the presence and severity of coronary atherosclerosis in patients undergoing coronary angiography

**DOI:** 10.1097/MD.0000000000030881

**Published:** 2022-09-30

**Authors:** Libo Yang, Mingliang Zhang, Hui Zhang, Guanlin Zheng, Chao Xu, Guangyao Li

**Affiliations:** a Department of Endocrinology, Shandong Provincial Hospital, Cheeloo College of Medicine, Shandong University, Jinan, Shandong Province, P.R. China; b Liaocheng People’s Hospital, Liaocheng, Shandong Province, P.R. China; c Department of Endocrinology, The Affiliated Taian City Central Hospital of Qingdao University, Taian city, Shandong Province, P.R. China; d Department of Cardiology, The Affiliated Taian City Central Hospital of Qingdao University, Taian city, Shandong Province, P.R. China; e Department of Clinical laboratory, The Affiliated Taian City Central Hospital of Qingdao University, Taian city, Shandong Province, P.R. China; f Taishan vocational college of nursing, Taian city, 271000, Shandong Province, P.R. China.

**Keywords:** coronary artery disease, Gensini score, thyroid autoimmunity

## Abstract

Studies on the association of thyroid autoimmunity with cardiometabolic risk and coronary artery disease (CAD) have produced conflicting results. This study aimed to investigate the relationship of thyroid autoimmune bodies (thyroid peroxidase antibody [TPOAb] and thyroglobulin antibody [TgAb]) with CAD in euthyroid subjects undergoing coronary angiography.

A total of 307 subjects who underwent coronary angiography were included. The severity of coronary atherosclerosis was evaluated by using Gensini score. Serum TSH, total T3, total T4, TPOAb, TgAb, lipid levels et al were measured and compared between the groups with and without CAD. Logistic multivariate regression analysis were performed to assess the associations. Levels of thyroid hormones were comparable between the two groups.

The positive percentage of anti-Tg antibodies was higher in non-CAD group (15.22% vs 7.91%, χ^2^ = 3.95, *p* = .047) while no significant difference was observed for anti-TPO antibodies (19.57% vs 17.21%, χ^2^ = 0.243, *p* = .622). The natural log-transformed Gensini score (ln (Gensini score)) was lower in the TgAb+ group (2.94 ± 1.11 vs 2.41 ± 1.18, P = .015). There was no significant difference for ln (Gensini score) between TPOAb− and TPOAb+ group (2.90 ± 1.14 vs 2.85 ± 1.09, P = .782). Logistical regression analysis revealed that positive TgAb was inversely associated with the presence of CAD (OR: 0.387, 95% CI: 0.157–0.952, *p* = .039) independent of other risk factors.

The results showed that TgAb positivity might be an independent protective factor for CAD.

## 1. Introduction

Thyroid autoimmunity, characterized by the production of thyroid autoantibodies and lymphocytic infiltration into the thyroid, is an archetypal organ-specific autoimmune disorder.^[1]^ The persistent presence of thyroid autoantibodies in the serum in titers greater than or equal to the upper level of normal interval represents the minimum criterion for the diagnosis of thyroid autoimmunity. Thyroid autoimmunity not only causes damage to thyroid tissues, but may also have potential extra-thyroidal actions.^[2]^ It was reported that thyroid autoimmunity per se was associated with some risk factors for atherosclerosis, including HbA1c, HOMA-IR, obesity, central obesity, hyperlipidemia, and metabolic syndrome.^[3]^

With respect to the specific autoantibodies of thyroid autoimmunity, thyroglobulin antibody (TgAb) and thyroid peroxidase antibody (TPOAb), their associations with cardiometabolic factors were explored by some studies,^[4-12]^ but with inconsistent results. Evidence on the relationship of thyroid autoantibodies with coronary artery disease (CAD) in euthyroid status is scarce. Therefore, we conducted this study to investigate the relationship of thyroid autoimmunity with the presence and severity of CAD in euthyroid subjects.

## 2. Methods

### 2.1 Subject

Subjects who underwent coronary angiography during the period from March 2020 to October 2021 were retrospectively reviewed. The exclusion criteria were as follows: Abnormal serum levels of TSH, TT4, or TT3; History of thyroid dysfunction or under treatment with thyroid medication; Acute or past myocardial infarction; History of revascularization of the coronary arteries; severe cardiac, hepatic or renal dysfunction; Medication with amiodarone, sex hormones and steroid hormones. Finally, a total of 307 subjects were included. The project was approved by the local Ethics Committee.

### 2.2 Data collection

Information on past medications and risk factors for CAD, including smoking, drinking, hypertension, and diabetes et al were obtained from medical records. Smokers and drinkers were defined as those who had smoked for at least 5 years and those with average daily alcohol consumption higher than 10 g for at least 5 years, respectively, regardless of whether they quitted or not. Diagnoses of diseases, including hypertension and diabetes mellitus, were based on the international Classification of Diseases, 10th Revision. BMI was calculated by dividing the weight (Kg) with the square of height (m^2^). Fasting venous blood specimens were collected to measure lipid profile, uric acid, creatinine, fibrinogen, thyroid hormones, TPOAb and TgAb.

 Coronary angiography was carried out via the radial artery or femoral artery. Coronary angiographic findings were reviewed by two expert cardiologists and significant stenosis was defined as a diameter stenosis of 50% or greater. We used the Gensini score^[15]^ to assess the severity of stenosis of coronary arteries: it scores it as 1 for 1% and 25% narrowing, 2 for 26% and 50%, 4 for 51% and 75%, 8 for 76% and 90%, 16 for 91% and 99%, and 32 for complete occlusion. This score is then multiplied by a factor, depending on the functional significance of the coronary artery. The multiplying factor for a left main stem lesion is 5. It is 2.5 for proximal left anterior descending artery (LAD) and left circumflex artery lesions, 1.5 for a mid-LAD lesion, and 1 for distal LAD, mid/distal left circumflex artery, and right coronary artery lesions. The multiplication factor for any other branch is 0.5.

 The lipid profile, uric acid, creatinine, and fibrinogen were measured by an automatic biochemistry analyzer (Modular DPP, Roche, Switzerland) and commercial kits.

The levels of TT3, TT4, TSH, and thyroid autoimmune bodies were measured by using the same chemiluminescence immunoassay among all subjects (ADVIA Centaur XP, Siemens). The laboratory reference ranges for TSH, TT3, TT4 were 0.35 to 5.5 mIU/L, 0.92 to 2.79 nmol/L, and 24.5 to 171.6 nmol/L, respectively. Cutoff value for TPOAb “+”or TgAb “+” was ≥60 IU/mL.

### 2.3 Statistical analysis

Data were represented as mean ± standard deviation. Inter-group analysis was done by using independent *t* test, Mann–Whitney *U* test, and chi-square test. Gensini score was natural log-transformed before analysis due to the obvious deviation from the normal distribution. Analysis of covariance was performed to adjust for age and sex. Multivariate binary logistic regression models were used to assess the association between thyroid autobodies and CAD. Statistical significance was set at *P* < .05. All above analyses were conducted with the use of SPSS software (SPSS Inc., Chicago, IL) in version 19.0.

## 3. Results

A total of 307 subjects meeting the inclusion criterion were finally included. The basic characteristics of all subjects and comparisons between CAD and non-CAD groups were displayed in Table 1. The mean age was 61.64 ± 10.38 of a year (age range: 30–82 years). A total of 70.03% of the patients presented CAD. Age and percentage of male and diabetes were significantly higher in CAD group compared with non-CAD group. The levels of lipids, total cholesterol (TC), triglyceride, low-density lipoprotein cholesterol, high-density lipoprotein cholesterol, creatinine, fibrinogen and uric acid were not significantly different between non-CAD and CAD group. Levels of TSH, TT4, and TT3 were similar between CAD and non-CAD group (TSH: 2.23 ± 1.16 vs 2.02 ± 1.08 mIU/L; TT4: 104.08 ± 27.79 vs 107.33 ± 30.34 nmol/L, TT3: 1.61 ± 0.29 vs 1.68 ± 0.38, respectively). With respect to thyroid autoimmunity, the positive percentage of TgAb was higher in non-CAD group (15.22% vs 7.91%, χ^2^ = 3.95, *P* = .047) while no significant difference was observed for TPOAb (19.57% vs 17.21%, χ^2^ = 0.243, *P* = .622).

**Table 1:** Clinical characteristics of total subjects.

Variable	Overall (n = 307)	Non-CVD (n = 92)	CVD (n = 215)	*p* value
Age (yr)	61.64 ± 10.38	59.10 ± 10.46	62.67 ± 10.19	.006
Gender (male %)	56.68	45.65	61.40	.011
BMI (kg/m^2^)	26.01 ± 3.71	25.68 ± 4.03	26.13 ± 3.56	.328
Smoking (%)	35.83	28.26	38.07	.070
Alcohol drinking (%)	27.36	21.74	29.77	.148
Diabetes (%)	21.82	10.87	26.51	.002
Hypertension (%)	53.42	46.74	56.28	.125
TC (mmol/L)	3.97 ± 0.99	4.05 ± 1.11	3.93 ± 0.94	.350
LDL-c (mmol/L)	2.73 ± 0.82	2.52 ± 0.79	2.49 ± 0.75	.770
TG (mmol/L)	1.62 ± 1.47	1.55 ± 1.46	1.65 ± 1.48	.607
HDL-c (mmol/L)	1.13 ± 0.29	1.18 ± 0.29	1.11 ± 0.29	.089
Creatinine (umol/L)	72.48 ± 14.47	71.08 ± 12.53	73.15 ± 15.24	.256
Uric acid (mmol/L)	305.44 ± 85.66	316.99 ± 83.20	300.83 ± 86.77	.135
Fibrinogen	3.11 ± 4.17	2.75 ± 0.58	3.27 ± 4.97	.327
TSH (mIU/L)	2.22 ± 1.18	2.23 ± 1.16	2.02 ± 1.08	.122
TT3 (nmol/L)	1.67 ± 0.35	1.61 ± 0.29	1.68 ± 0.38	.081
TT4 (nmol/L)	106.38 ± 29.51	104.08 ± 27.79	107.33 ± 30.34	.377
Anti-TPO (%)	17.92%	19.57	17.21	.622
Anti-TG (%)	10.10%	15.22	7.91	.047

BMI = body mass index, HDL-c = high density lipoprotein-cholesterol, LDL-c = low density lipoprotein-cholesterol, TC = total cholesterol, TG = triglyceride, TSH = thyroid stimulating hormone.

**Figure 1.**
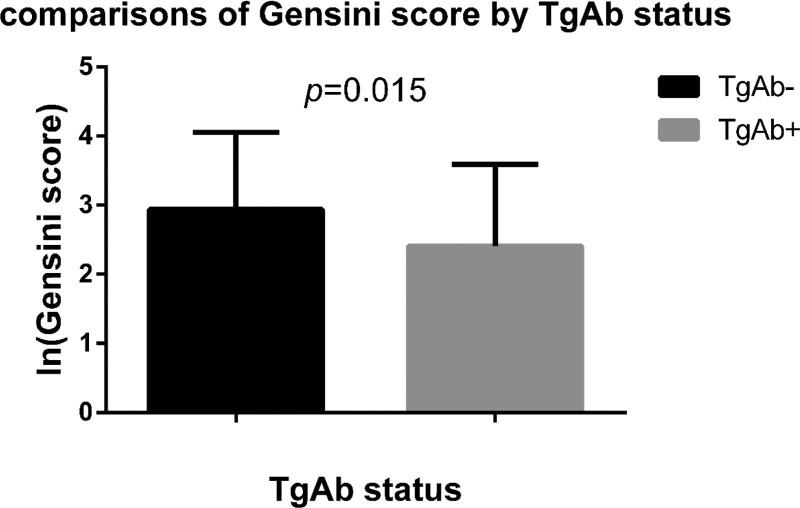
Comparisons of ln (Gensini score) between TgAb− and TgAb+ group. TgAb = thyroglobulin antibody.

When comparing with the TgAb+ group, ln (Gensini score) was higher in TgAb− group (2.94 ± 1.11 vs 2.41 ± 1.18, P= 0.015) (Fig. 1), while there was no significant difference of ln (Gensini score) between TPOAb− and TPOAb+ group (2.90 ± 1.14 vs 2.85 ± 1.09, P = .782).

Multiple logistic regression analysis showed positive TgAb was an independent protective factor for CAD, with an adjusted odds ratio (OR) of 0.387 (95% CI: 0.157–0.952, *P* = .039). Other independent factors for predicting CAD included age (OR: 1.053, 95% CI: 1.021–1.087, *P* = .001), gender (OR: 0.369, 95% CI: 0.141–0.962, *P* = .041), diabetes (OR: 3.367,95% CI: 1.399–8.100, *P* = .007), uric acid (OR: 0.995, 95% CI: 0.991–0.999, *P* = .016) and low density lipoprotein-cholesterol (OR: 2.441, 95% CI: 1.063–5.608, *P* = .035). TPOAb was not significantly associated with CAD (OR: 0.797, 95% CI: 0.387–1.642, *P* = .539) (Table 2).

**Table 2:** Multiple logistic regression analysis for CVD.

Variables	Odds ratio	95%CI	*p* value
Age	1.053	1.021–1.087	.001
Gender	0.369	0.141–0.962	.041
Smoking	0.973	0.396–2.393	.953
Alcohol drinking	1.065	0.455–2.496	.884
BMI	1.052	0.965–1.147	.250
Diabetes	3.367	1.399–8.100	.007
Hypertension	0.894	0.485–1.648	.720
TC	0.582	0.274–1.239	.160
TG	1.232	0.934–1.625	.139
LDL-c HDL-c	2.441 0.923	1.063–5.608 0.217–3.932	.035 .914
Creatinine Fibrinogen	1.006 1.568	0.980–1.032 0.939–2.619	.672 .086
Uric acid	0.995	0.991–0.999	.016
TSH	0.969	0.745–1.295	.812
TT4	0.993	0.981–1.006	.293
TT3	2.601	0.794–8.520	.114
TPOAb	0.797	0.387–1.642	.539
TgAb	0.387	0.157–0.952	.039

BMI = body mass index, HDL-c = high density lipoprotein-cholesterol, LDL-c = low density lipoprotein-cholesterol, TC = total cholesterol, TG = triglyceride, TPOAb = thyroid peroxidase antibody, TSH = thyroid stimulating hormone.

## 4. Discussion

Our present study on euthyoid patients undergoing coronary angiography revealed that TgAb was inversely associated with the presence of CAD after adjustment for confounding factors. Furthermore, we found that the subjects with the presence of TgAb had less severe stenosis of the coronary arteries, which assessed by the Gensini score. However, we did not find a significant association of TPOAb with CAD and the severity of stenosis of coronary arteries.

The association of thyroid autoimmunity with cardiovascular diseases has been explored by some studies among subjects with thyroid dysfunction. Most of these studies did not find a significant association between thyroid autoimmunity and cardiovascular diseases,^[13-17]^ except two studies.^[18, 19]^ The study by Hak AE et al^[19]^ showed an increased odds ratio for aortic atherosclerosis and a history of myocardial infarction with the presence of positive TPOAb in subclinical hypothyroidism. However, no association was found between thyroid autoimmunity itself and cardiovascular disease in the study by Hak AE et al Another study^[18]^ identified that TPO-Ab detectability was associated with a higher risk of cardiovascular mortality in 9685 participants. Contrary to these findings, our study only observed a significant relationship of TgAb with the presence and severity of CAD, but not for TPOAb. The reasons for the discrepancies included the different thyroid status and study population. The above-mentioned studies included the subjects with thyroid dysfunction. However, only euthyroid subjects were enrolled in our study to exclude the role of thyroid hormones and examine the effects of thyroid autoimmunity itself on CAD. Besides, our study was performed among patients undergoing coronary angiography, which can ensure a more accurate diagnosis of CAD.

The underlying mechanisms involved in the inverse association between TgAb with CAD remain uncertain. Some studies have shown a protective role of TgAb for metabolic disorders in the euthyroid population. A national epidemiological survey demonstrated that serum TgAb single positivity was an independent protective factor for impaired fasting blood glucose and hypertriglyceridemia in euthyroid general population.^[5]^ Another study reported an independently negative association between abnormal TgAb levels and metabolic dysfunction-associated fatty liver disease among 424 euthyroid patients.^[4]^ The protective role of TgAb in metabolic disorders has been speculated to be related to carbonic anhydrase (CA) activity. Decreased CA activity may induce the production of TgAb via high levels of iodine uptake.^[20]^ Studies have identified the role of CA in obesity and nonalcoholic fatty liver disease, both of which are risk factors for CAD.^[21, 22]^ Therefore, the protective effect of serum TgAb positive expression on CAD may be due to potentially abnormal CA activity. However, these speculations and elucidation of the exact mechanism require further studies.

 The investigation on the role of TPOAb in CAD risk factors among euthyroid subjects revealed discordant results. Several studies showed that TPOAb was positively associated with HOMA-IR and hsCRP levels,^[7]^ endothelial dysfunction,^[8]^ intima media thickness,^[9, 23]^ homocysteine,^[9]^ and hypertension.^[24]^ However, two other studies generated nonsignificant and even opposite results. Agbaht K et al reported that TPOAb levels had no association with metabolic syndrome among 584 subjects,^[10]^ and a negative relationship of TPOAb with metabolic syndrome and its triglyceride component was observed by Raposo, L et al in a subsample of 486 participants.^[6]^ These conflicting results indicated that the role of TPOAb in CAD risk needed further confirmation. Therefore, it is not surprising that the correlations were null between TPOAb and CAD in our study.

Several limitations of this study should be considered. First, because of the cross-sectional nature of our design, the direction of any causal relationship could not be established. Prospective studies are needed to confirm our results. Second, although many well-known factors associated with CAD were analyzed in our study, residual confounding cannot be completely adjusted for. For example, family history was not collected in our study. However, both CAD and autoimmune thyroid disease have a genetic basis and certain degree of family clustering. Family history may be one of the residual confounders. Therefore, we cannot definitively exclude that the significant associations between thyroid autoimmunity and CAD could be, at least partially, explained by some of these unmeasured factors. Finally, given thyroid function is influenced by many nonthyroidal illnesses and drugs,^[25,26]^ only single measurement of thyroid hormones may result in misclassification.

In conclusion, our study showed that TgAb was inversely associated with the presence and severity of CAD among euthyroid subjects, implying that TgAb positivity might be a protective factor for CAD. The underlying mechanisms related to these interesting phenomena await further investigation through basic research work.

## Author contributions

Conceptualization: Chao Xu.

Data curation: Mingliang Zhang, Hui Zhang, Guanlin Zheng.

Formal analysis: Chao Xu, Libo Yang, Hui Zhang.

Investigation: Libo Yang, Mingliang Zhang, Hui Zhang, Guanlin Zheng.

Supervision: Chao Xu, Guangyao Li.

Validation: Mingliang Zhang.

Writing – original draft: Libo Yang.

Writing – review & editing: Chao Xu, Guangyao Li.
